# Renal abnormalities among HIV-infected, antiretroviral naive children, Harare, Zimbabwe: a cross-sectional study

**DOI:** 10.1186/1471-2431-13-75

**Published:** 2013-05-11

**Authors:** Vongai Dondo, Hilda A Mujuru, Kusum J Nathoo, Maxwell Chirehwa, Zivanai Mufandaedza

**Affiliations:** 1 Department of Paediatrics and Child Welfare, University of Zimbabwe, College of Health Sciences, Avondale, Harare, Zimbabwe; 2 Department of Community Medicine, University of Zimbabwe, College of Health Sciences, Avondale, Harare, Zimbabwe; 3 Department of Medical Laboratory Sciences, University of Zimbabwe, College of Health Sciences, Avondale, Harare, Zimbabwe

## Abstract

**Background:**

Data on the prevalence of renal and urine abnormalities among HIV-infected children in Sub-Saharan Africa are limited. We set out to determine the prevalence of proteinuria; low estimated glomerular filtration rate (eGFR), urinary tract infection and associated factors among HIV-infected antiretroviral therapy (ART) naive children, aged 2–12 years, attending the paediatric HIV clinic at a tertiary hospital in Harare.

**Methods:**

Consecutive ART naive children attending the clinic between June and October 2009 were recruited. Detailed medical history was obtained and a complete physical examination was performed. Children were screened for urinary tract infection and for significant persistent proteinuria. Serum creatinine was used to estimate GFR using the modified Counahan-Barratt formula. The Student’s t-test was used to analyse continuous variables and the chi-square or Fisher’s exact test was used to analyse categorical data. Logistic regression was performed to assess the relationship between study factors and urine abnormalities, persistent proteinuria and the eGFR.

**Results:**

Two hundred and twenty children were enrolled into the study. The median age was 90 months (Q1=65.5; Q3=116.5). The prevalence of urinary tract infection was 9.5%. Escherichia coli was the predominant organism. There was uniform resistance to cotrimoxazole. Persistent proteinuria (urine protein to creatinine ratio greater than 0.2, a week apart) was found in 5% of the children. Seventy-five children (34.6%) had mild to moderate renal impairment shown by a low eGFR (30 to <90ml/min/1.73m2). Persistent proteinuria was more likely to be found in children who were wasted, weight-for-height (WHZ) z-score <−2 (p=0.0005). Children with WHO clinical stage 4 were more likely to have a low eGFR than children with less advanced stages (OR 2.68; CI 1.24-5.80). Urine abnormalities were more likely to be observed in children with WHO clinical stages 3 and 4 (OR 2.20; CI 1.06-4.60).

**Conclusion:**

There is significant renal impairment among HIV-infected, ART naive children aged 2–12 years attending the outpatient paediatric HIV clinic at Harare Central Hospital. The abnormalities are more likely to occur in children with advanced HIV/AIDS. Screening for renal impairment and urinary tract infections in HIV-infected children before initiation of ART and regularly thereafter would be helpful in their management. Keywords: HIV, renal disease, persistent proteinuria, glomerular filtration rate, urinary tract infection

## Background

Almost 30 million HIV-infected people live in Africa and Sub-Saharan Africa is the single most affected region. Sub-Saharan Africa has 10% of the world’s population, yet about 60% of the world’s HIV-infected people live in the region. It is estimated that 300 000 HIV-infected children living in the Sub-Saharan Africa could develop HIV-1 associated nephropathy (HIVAN) if they do not receive appropriate antiretroviral therapy (ART) [1,2].

The prevalence of renal diseases in HIV-infected children before the antiretroviral therapy era in the USA was estimated to be 40% [3-6]. Since the widespread establishment of ART in 1996, the clinical outcome of HIV-infected children in the United States has improved dramatically and HIVAN is being diagnosed at an older age [2]. A study in India by Shah et al. showed that 53.6% had renal manifestations with abnormal glomerular filtration rate in 44% of the HIV-infected, highly active antiretroviral naive children [7]. In Sub-Saharan Africa, there are a few studies which describe the extent of renal disease in HIV-infected children. In Nigeria, Iduoriyekemwen et al. found the prevalence of renal disease in HIV-infected children on highly active antiretroviral therapy (HAART) to be 16.2% [8]. Some of the studies in sub-Saharan Africa focused on proteinuria since it has been shown to be an early marker for HIVAN. Ekulu et al. described the prevalence of proteinuria and its association with HIV/AIDS in Congolese children living in Kinshasa [9]. A similar study was done among HIV-infected children attending a tertiary hospital in Lagos and Port Harcourt in Nigeria [10,11]. Recent studies in Sub-Saharan Africa report on proteinuria as an early marker of HIVAN and some include the prevalence of proteinuria and renal disease in children on HAART [8-11]. It is anticipated that with the increased availability of HAART, the knowledge in diagnosing and treating renal disease in HIV-infected children will improve.

There is a wide clinical spectrum of renal disease in the course of HIV infection [12,13]. The mechanism of kidney involvement in HIV infection is multifactorial. Opportunistic infections, drugs used in the treatment of HIV and its complications or malignancies associated with advanced immunosuppression may contribute to renal disease in HIV-infected individuals [13]. Acute and chronic kidney injury have been described and the causes are similar to HIV-uninfected children [6,13]. Various electrolyte disorders particularly hyponatraemia have been documented in hospitalized HIV-infected children [13]. Asharam et al. in South Africa found no significant impact of HIV/AIDS on the presentation of bacterial urinary tract infection in HIV-infected children aged 0–12 years, similar to findings by O’Regan et al. in Canada [14,15]. In Nigeria, Ibadin et al. found an increased frequency of UTI in adolescent and young adult patients with advanced WHO clinical stages [16].

HIV-associated nephropathy (HIVAN) has been described as the most common renal abnormality among HIV-infected adults in the black populations in the USA. It was initially described in adults in 1984 and in children shortly thereafter [17,18]. HIVAN is associated with nephrotic range proteinuria, hypoalbuminaemia and normal or low cholesterol levels [14,19-22]. Various mechanisms of glomerular injury in HIV infection have been described [5,13]. HIVAN may occur early on during the course of HIV-1 infection. The human immunodeficiency virus itself may cause kidney damage. The viral genes *env, tat, nef and vpr* have been linked to the pathogenesis of renal disease [23]. Renal parenchymal cells can serve as a reservoir of HIV and the presence of the virus can persist in the tubular and glomerular epithelial cells despite antiretroviral therapy. Entry into epithelial cells is via CD4 and CXCR4 receptors. However, HIV DNA has been found in the glomeruli of HIV infected patients without HIVAN suggesting that other factors come into play e. g genetics [18]. Recently, Gu et al. reported that expression of the signal transducer and activator of transcription (STAT) 3 is necessary for the full manifestation of the HIVAN phenotype in mice [24]. Other mechanisms include damage from circulating immune complexes, direct cytopathogenic effect on glomerular cells with undefined mechanisms, haemodynamic disturbances and nephrotoxicity of drugs used in the treatment of HIV and related illnesses [19]. Focal segmental glomerulosclerosis with microcystic tubular dilatation and mesangial hyperplasia have been documented with almost equal frequency in children [18,25]. The development of end stage renal disease was noted to be slower in children than in adults [18,20-22].

This study aimed to document the prevalence of renal impairment, urine abnormalities and the associated factors among HIV-infected, antiretroviral naive children in a resource-limited setting. This study reports the prevalence of and factors associated with persistent proteinuria, a low eGFR and urinary tract infection among HIV-infected children attending the HIV clinic.

## Methods

### Study design and study participants

The cross sectional study was carried out at the paediatric HIV clinic at Harare Hospital, Zimbabwe between June and October 2009. The hospital serves mostly the lower socio-economic group within the city, peri-urban and surrounding rural areas. The clinic had just over 3 000 HIV-infected children registered, with about 2000 of them being antiretroviral naïve at the commencement of the study. The majority of these children have vertically transmitted HIV infection. Ferrand et al. reported a very low (1.3%) herpes simplex virus (HSV)2 seropositivity in HIV-infected adolescents admitted at the same hospital and another tertiary hospital in Harare. This suggests mother-to-child transmission as their source of infection since HSV-2 infection is a highly prevalent sexually transmitted infection in southern Africans with sexually acquired HIV infection [26]. ART naïve children, aged 2–12 years who attended the clinic during the study period were eligible for enrolment. Children with known risk factors for renal disease e.g. diabetes mellitus, sickle cell disease, hypertension, or acutely ill children requiring hospitalization were excluded.

Consecutive eligible children attending the clinic were recruited and subsequently enrolled into the study after caregivers provided written informed consent and children 7 years and older provided written informed assent.

The study was approved by the Institutional Review Board and the Medical Research Council of Zimbabwe.

### Clinical assessment

Clinical history and complete physical examination including blood pressure measurement were done at enrolment. Weights and heights were measured and WHO 2007 growth charts were used as reference for determining the nutritional status. Blood pressure was considered normal if it was less than 95^th^ centile for age, sex and height. The participants were clinically staged using the clinical and immunological system recommended by WHO (2006) [27].

### Laboratory assessment

A midstream urine sample was collected for dipstick analysis, microscopy, culture and sensitivity. The urine samples were analyzed immediately using a dipstick (strips by Siemens Healthcare Diagnostics, United Kingdom). Proteinuria on dipstick was reported as negative, 1+ (30 mg/dL), 2+ (100 mg/dL), 3+ (300 mg/dL) or 4+ (1000 mg/dL) [28]. Participants had a repeat urine dipstick analysis after 1 week if proteinuria was 1+ or more in a urine specimen with specific gravity <1.015 or proteinuria 2+ or more in urine specimen with a specific gravity of ≥1.015 [29]. If repeat urine dipstick analysis showed persistent proteinuria after 1 week, it was considered significant and the urine specimen was sent for random spot urine protein/creatinine (Upr/cr) ratio for semi-quantification [29,30]. These were measured using the Synchron CX5 Delta clinical system (Beckman Instruments, Inc. Fullerton USA). Urine protein concentration was measured by the timed end point method and urine creatinine concentration was measured by the modified Jaffe method [31,32]. Persistent proteinuria was defined as urine protein/creatinine ratio >0.2, further classified into intermediate proteinuria (0.2-3.0) and nephrotic range proteinuria (>3.0) [30]. The presence of orthostatic proteinuria was not assessed.

All urine specimens were kept refrigerated between +2°C and +8°C until processing, which was done within 5 hours [30,31]. Specimens were analyzed at the University of Zimbabwe laboratories. Urine culture was done by half plate isolation technique using a 1/1000 (1 μL) urine-inoculating loop. Two culture media were used, the Cystine Lactose Electrolyte Deficient (CLED) and blood agar, incubating the plates aerobically at 37°C overnight [32]. A growth of >10^5^ organisms was considered significant. Gram stain and biochemical tests were done to identify the organisms. Antimicrobial sensitivity testing was done on Mueller Hinton Agar using the standard Kirby Bauer Technique with antibiotic sensitivity discs (Mastrings Diagnostics). Sensitivity was reported using standard zones of inhibition.

Blood samples were collected for serum creatinine from all the children and for CD4^+^ T-cell (CD4) count only in those who had CD4 count done more than 4 weeks prior to enrollment into the study. Serum creatinine was measured using the modified Jaffe reaction [32,33]. An estimation of the GFR was by the modified Counahan-Barratt formula expressed as milliliters per minute per 1.73 meter’ squared (ml/min/1.73 m^2^) [33].The modified Counahan-Barratt method was used rather than the Schwartz because serum creatinine was measured using the modified Jaffe reaction. When serum creatinine is estimated by the modified Jaffe reaction rather than by the enzymatic method, noncreatinine chromogens generate sufficient colour to account for about 30μmol/dL of creatinine. The Schwartz formula tends to overestimate GFR in this case. The Counahan-Barratt formula adjusts for the noncreatinine chromogens and gives a more accurate value of creatinine and GFR estimate [33,34]. Renal dysfunction as indicated by a low eGFR was classified as mild (60-89 ml/min/1.73 m^2^), moderate (30-59 ml/min/1.73 m^2^), and severe impairment (15-29 ml/min/1.73 m^2^) [35].

### Data analysis

Data were analyzed using STATA 10.0. Continuous variables were described as means and compared by the Student’s *t-*test. Categorical data was described as proportions and analyzed using chi-square or Fisher’s exact test. Logistic regression was performed to assess the relationship between study factors and urine abnormalities, persistent proteinuria and eGFR. A p value ≤ 0.05 was considered significant. Odds ratios are also presented.

## Results

Two hundred and twenty children were enrolled with a male to female ratio of 1:1.2. The median age was 90 months (Q_1_=65.5; Q_3_=116.5). Ninety-eight (45.6%) children were underweight. One hundred and thirty children (59%) children were stunted while 4.5% and 3.6% were moderately wasted (WHZ between −2 and −3) and severely wasted (WHZ <−3) respectively. Eight children were stunted and moderately wasted while 5 children were stunted and severely wasted.

### WHO HIV staging of the children

Forty children (18.2%) were clinical stage 1; 61 (27.7%) stage 2, 88 (40%) stage 3 and 31 (14.1%) stage 4. One hundred and ninety eight children (90%) had available CD4 counts. Using the WHO immunological staging, 82 (41.4%) children were not significantly immunosuppressed, 35 (17.7%) had mild immunosuppression, 35 (17.7%) advanced immunosuppression and 46 (20.1%) severe immunosuppression (Additional file 1). Of the 111 children with clinical stages 3 and 4, 56 (50%) had corresponding advanced to severe immunosuppression.

### Proteinuria

Dipstick analysis was performed on 220 urine specimens. Proteinuria of 1+ or more was detected on 36/220 (16%) specimens. Nineteen of the 36 children met the criteria for repeat urine dipstick analysis. Seventeen of these 19 children (89%) came for repeat urinalysis and 11 had persistent proteinuria. These specimens were sent for urine protein and creatinine quantitation. The Upr/cr in the 11 samples was greater than 0.2, giving a prevalence of persistent proteinuria of 11/220 (5%). Six of the 11 children had intermediate proteinuria while 5 children had nephrotic range proteinuria. Only 2 children with persistent proteinuria had oedema (one had facial oedema only and one had bilateral pitting pedal oedema). One child with persistent proteinuria had a raised diastolic blood pressure. Six children with persistent proteinuria also had haematuria on dipstick analysis and 3 of these children had the haematuria confirmed on microscopy. Four of the 11 children with persistent proteinuria had reduced eGFR.

Persistent proteinuria was more likely to occur in children with WHZ <−2 compared to children with WHZ ≥−2 [p=0.0005) (Table 1)]. Stunting and underweight did not have a significant effect on persistent proteinuria.

**Table 1:** Factors associated with persistent proteinuria among HIV-infected, ART naïve children, 2–12 years old (n=220)

**Variable**	**Persistent proteinuria n (%)**	**OR**	**95% CI**	**P value**
Median age in months (Q1;Q3)	106 (86;121)	1.01	0.99-1.04	0.175
Gender males	6 (5.9)	0.69	0.21-2.35	0.557
females	5 (4.2)			
WAZ ≥−2	3 (4.1)	3.36	0.82-13.63	0.09
<−2	8 (12.5)			
WHZ ≥−2	7 (3.6)	**7.96**	**1.71-35.87**	**0.0005**
<−2	4 (22.2)			
eGFR(ml/min/1.73m^2^)				
≥90	7 (4.9)	1.09	0.31-3.84	0.897
<90	4 (5.3)			
Diastolic BP normal	10 (5.2)	1.4	0.17-11.80	0.757
high	1 (7.1)			
Clinical stages 1&2	1 (1.0)	**9.17**	**1.15-72.96**	**0.036**
Clinical stages 3&4	10 (8.4)			
Immunological stages				
Not significant	3 (3.7)	-	-	-
Mild	2 (5.7)	1.60	0.25-10.0	0.618
Advanced	3 (8.6)	2.47	0.47-12.88	0.284
Severe	3 (6.5)	1.84	0.36-9.50	0.468

WAZ- Weight-for-age z-score.

WHZ- Height-for-age z-score.

Logistic regression and odds ratios.

### Glomerular filtration rate

An estimation of the GFR was calculated using the modified Counahan-Barratt formula in 217 children. The eGFR was ≥90 ml/min/1.73 m^2^ in 142 (65.4%). Seventy-five children had a low eGFR with 73 (33.6%) having mild renal impairment and 2 (1%) having moderate renal impairment. Serum creatinine was within the normal range in 73 of 75 (97%) children who had a low eGFR. Younger children (median age 77months) were less likely to have a low eGFR compared to slightly older children (median age 98months) (OR 0.98; CI 0.97-0.99). Children with WHO clinical stage 4 were more likely to have a low eGFR than children with less advanced stages [(OR 2.68; CI 1.24-5.80) (Table 2)].

**Table 2:** Factors associated with a low eGFR among HIV-infected, ART naïve children, 2–12 years old (n=217)*

**Variable**	**eGFR <90 ml/min/1.73 m**^**2**^	**OR (95% CI)**	**p-value**
Median age (Q1;Q3)months	77 (57;103)	**0.98 (0.97; 0.99)**	**<0.001**
Gender Males	35 (35.4)		
Females	40 (33.9)	0.94 (0.54; 1.64)	0.822
WAZ ≥−2	36 (55)		
<−2	39 (40)	1.57(0.86; 2.87)	0.116
**Diastolic Blood Pressure**			
Normal	61(32.3)		
Low	5 (45.5)	1.75 (0.51; 5.95)	0.371
High	8 (57.1)	2.80 (0.93; 8.42)	0.067
**Clinical stages**			
Stage 1-3	58 (31.2)		
Stage 4	17 (54.8)	**2.68 (1.24; 5.80)**	**0.012**
**Immunological stage**			
Not significant	29 (36.3)		
Mild	12 (35.3)	0.96 (0.42; 2.22)	0.922
Advanced	7 (20.0)	0.44 (0.17; 1.13)	0.088
Severe	20 (43.5)	1.35 (0.65; 2.84)	0.424

^*^3 children did not have serum creatinine results and eGFR was not calculated.

Logistic regression done, odds ratios presented.

### Urinary tract infection

Bacterial urinary tract infections were diagnosed in 21 (9.5%) children (Table 3). The symptoms of UTI in these children were urinary frequency (10), dysuria (8), fever (5), loin pain (4), abnormal urine appearance (‘milky’ urine or thick urine) (3), macroscopic haematuria (2) and urgency (2). Eighteen children had more than one symptom. Fever was more likely to occur in children with a UTI than those without a UTI (p=0.0002).

**Table 3:** Urine abnormalities among HIV-infected, ART-naïve children 2-12years old

**Abnormality**	**Frequency (n=220)**	**%**
Urinary tract infection (bacterial)	21	9.5
Sterile pyuria	12	5.5
Asymptomatic bacteriuria	12	5.5
Haematuria (microscopy)	12	5.5
S.haematobium	3	1.4
Urinary casts	16	7.3
White cell casts	9	4.1
Hyaline casts	3	1.4
Epithelial casts	3	1.4
White and red cell casts	1	0.5
None	158	71.8

Of the 12 children with haematuria on microscopy half of them had a UTI (including 3 who had schistosomiasis). Sixteen children had urinary casts with only 5 of them having UTI (Table 3). Among the children with no UTI, 2 had hyaline casts and 3 had white cell casts. One child had both red cell and white cell casts. Crystalluria was more likely to be found in children with a UTI (4/21) compared those without (5/199) (p=0.005).

Thirty-three (15%) urine specimens yielded growth on culture. The antimicrobial sensitivity pattern (Table 4) shows that most of the organisms were sensitive to gentamicin and ciprofloxacin. There was uniform resistance to cotrimoxazole. The odds of urine abnormalities were higher in children with WHO clinical stages 3 and 4(OR 2.20; CI 1.06-4.60) (Table 5). Haematuria was more likely to be present in children with UTI than children without UTI (p=0.020). Proteinuria of 1+ or more was more likely to be present in children with a UTI (8/21) compared to children without UTI (28/199) (p=0.006). Leucocytes were positive in 7 children with a bacterial UTI. Nitrite was strongly positive in 1 child who had a UTI due to *E.coli*.

**Table 4:** Antimicrobial sensitivity pattern among the organisms isolated on culture n=33

**Drug**	***E. coli n=21***	***S. aureus n=5***	***Klebsiella n=3***	***Proteus n=2***	***β haemolytic Streptococcus n=3***
**Number of organisms sensitive to the drug**					
Gentamicin	13	4	3	1	3
Ciprofloxacin	17	5	3	2	3
Nalidixic acid	11	2	1	0	0
Tetracycline	2	0	1	0	1
Nitrofurantoin	5	0	0	0	1
Ampicillin	0	0	0	0	1
Cotrimoxazole	0	0	0	0	0

**Table 5:** The association between WHO HIV stages and urine abnormalities** on microscopy and culture among HIV-infected, ART naïve children, 2-12years old n=220

	**Urine abnormalities n (%)**	**OR (95% CI)**	**P value**
**Clinical stages**			
Stage 1&2	15 (12.9)		
Stage 3&4	33 (27.0)	**2.20(1.06; 4.60)**	**0.021**
Immunological stage***			
Not significantly immnosuppressed	11 (12.2)		
Mild immunosuppression	15 (42.9)	**5.4 (2.11; 13.84)**	**<0.001**
Advanced immunosuppression	8 (20.0)	1.8 (0.62; 5.20)	0.277
Severe immunosuppression	11 (23.9)	2.26(0.88; 5.83)	0.277

OR= Odds ratio CI=confidence interval.

**The urine abnormalities are UTI (including schistosomiasis), sterile pyuria and asymptomatic bacteriuria.

***Two children with urine abnormalities did not have CD4 count results.

Logistic regression and odds ratios presented.

## Discussion

This study shows a high prevalence of renal and urine abnormalities among HIV-infected ART-naïve children attending the paediatric HIV clinic at Harare Children’s hospital, with a low eGFR in 34% and UTIs in 10.9%. Eleven children had persistent proteinuria probably indicating underlying renal disease.

### Proteinuria

Proteinuria on the first urine dipstick analysis was found in 36 (16.4%), which is lower than what was found by Galgallo in Kenya (30%) in a similar age group (18months to 12 years) [36]. The Kenyan study included in-patients with two thirds of them having advanced HIV/AIDS disease and this could explain the higher prevalence of proteinuria. Two other studies in HIV-infected children showed a prevalence of proteinuria of 23.8% in Congolese children and 20.5% in Nigerian children [9,10]. However in these two studies, persistent proteinuria was not determined. The prevalence of microalbuminuria was found to be 12% in HIV-infected children on HAART in Nigeria [11]. Similarly, in ART naïve children in India the prevalence of persistent proteinuria was 10.7% [7]. The prevalence of persistent proteinuria in this study was 5%, similar to what Wools-Kaloustian et al. found in Kenyan adults (6%) under similar study settings [37]. However the prevalence is much lower than what Chaparro et al. reported in HIV-infected children in the USA (33%). Only 15% were ART-naïve and a lower cut off for nephrotic range proteinuria Upr/cr ratio >1.0 was used [17]. In this study urine dipstick analysis was done on all children and urine for protein/creatinine ratio was only done in those with persistent proteinuria. Chaparro et al. used a more precise test [29] by performing a urine protein/creatinine ratio in all the patients to detect proteinuria [17]. The use of the urine protein to creatinine ratio has been shown to have a high correlation with protein excretion determinations [30,38]. The best correlates were seen if the spot urine for protein and creatinine was done with the second voided urine after waking up [38].

Persistent proteinuria has been shown to be the earliest and consistent marker of HIVAN. The presence of persistent proteinuria could be an early manifestation of renal disease in our children though confirmation with a kidney biopsy is recommended. This is not always available in resource-limited settings. The median age of the children with persistent proteinuria in this study was 8.3 years (Q_1_=7.2; Q_3_=10.1). The mean age at diagnosis of HIVAN by Chaparro et al. was similar though most of their children were on ART and in their setting; urinalysis is done routinely on all HIV-infected children allowing earlier detection of proteinuria. Eke et al. also showed that microalbuminuria was more likely to occur in older children, with clinical AIDS and not on HAART [11]. With the widespread use of ART in the United States, HIVAN is being described at an older age and more HIV-infected children are surviving and requiring dialysis and transplantation [2].

Oedema has been shown to be minimal or absent in HIVAN because of hypergammaglobulinaemia which will maintain oncotic pressure. Two of the patients with persistent proteinuria in this study had oedema. The blood pressure measurements of the patients with persistent proteinuria were normal except in one patient who had a high diastolic blood pressure. Blood pressure in HIVAN has been described as normal and if raised, it is recommended that other causes of nephropathy be excluded [2,13,22]. Persistent proteinuria was significantly associated with haematuria detected by dipstick (p=0.004) and microscopy (p=0.022) similar to findings by Chaparro et al. suggesting glomerular damage that occurs in HIVAN [17].

Persistent proteinuria was more likely to occur in children with advanced WHO clinical stage as shown by other studies [9,10,17,19] although in this study the confidence interval was too wide, it could be due to small numbers of children with proteinuria. There was no association between immunological stage and persistent proteinuria in this study though other studies found that persistent proteinuria was more likely to occur at lower CD4 counts [17,19,40,41]. Four children with persistent proteinuria had a low eGFR. This was not statistically significant although Chaparro et al. reported significant association [17]. Small numbers of children with persistent proteinuria is a limitation. Children with a weight for height <−2SD were significantly more likely to have persistent proteinuria than well-nourished children. Duncan et al. in Jamaica showed that in the 6 children, who met the criteria of HIVAN, 4 had failure to thrive and one of these 4 children was stunted [41]. The failure to thrive or wasting may be a manifestation of the underlying chronic renal disease and or advanced HIV disease.

The prevalence of renal impairment in this study as indicated by a low eGFR was 34.6%. There was no significant association between renal impairment and sex, CD4 count or severe wasting but there was statistically significant association with advanced WHO clinical stage (p=0.012) similar to findings by Galgallo [36]. Young age was more likely to be associated with normal eGFR. The association with advanced clinical stage and older age suggests the occurrence of renal abnormalities as late manifestations of HIV [11]. Serum creatinine was normal in 97% of children with low eGFR consistent with the fact that serum creatinine may be an insensitive measure of decreased renal function and its level does not rise above normal until the GFR falls by 30-40% [29].

### Urinary tract infection

The prevalence of bacterial UTI in this study was 9.5%, higher than what has been documented in non HIV-infected children [29,30]. The prevalence of UTI in this study is about half of what Duncan et al. found (16.8%) among HIV-infected children in Jamaica. In the Jamaican cohort some children were on antiretroviral therapy although the exact proportion is not stated. Further evaluation revealed no structural abnormalities in children below 5years in the Jamaican cohort [41]. Ibadin et al. reported the prevalence of UTI of 6.3% in HIV-infected adolescents and adults with WHO clinical stages 1 and 2 [16]. UTI may be diagnosed with increased frequency in HIV-infected children partly due to contamination related to difficulties in collecting a representative specimen due to persistent diarrhoea which is a common complication of HIV infection [5].

Dysuria, urinary frequency, loin pain, urgency and abnormal urine appearance were the common symptoms, similar to findings by Ibadin et al. [16]. These are similar to non HIV-infected children [14]. In this study, fever was more likely to occur in children with UTI than those without as shown by Ibadin et al. [16]. UTI should therefore be considered in HIV-infected children presenting with fever. In this study, 5.5% of the children had asymptomatic bacteriuria similar to the findings by Ibadin et al. [16]. Although asymptomatic bacteriuria is considered benign, the high prevalence found in our study may need further exploration.

The commonest organism isolated was *E.coli* followed by *S. aureus, Klebsiella* and *Proteus* (Table 6) similar to findings in other studies [16,29,30,34]. *E. coli* is the causative agent in about 80-90% cases of UTI followed by *Klebsiella* and *Proteus*[29,30]. *S. aureus* was the causative organism in 4 children with UTI and has been described as a causative organism for UTI in other studies [19,34]. Where there is malformation or dysfunction of the urinary tract, less virulent organisms such as *Pseudomonas, Staphylococcus aureus* or *epidermidis* or *Group B Streptococcus* may cause a UTI [34]. However other studies found uropathogens such as Salmonella species, *Acinetobacter calcoaticus,* Enterococci, and *Pseudomonas aeruginosa*[34,41] that require specialized media for growth and could not be cultured in this study.

**Table 6:** Aetiological agents for culture proven urinary tract infections n=33

**Organism**	UTI*(n=21)	**Asymptomatic bacteriuria (n=12)**
*E. coli*	15	6
*S.aureus*	4	1
*Klebsiella*	1	2
*β haemolytic Streptococcus*	1	2
*Proteus*	1	1

*One specimen grew both *E.coli* and β *haemolytic Streptococcus.*

The majority of the E *coli* isolates were sensitive to ciprofloxacin 17/21(80.9%) while 61% and 52.3% were sensitive to gentamicin and nalidixic acid respectively. *E. coli* was resistant to ampicillin and cotrimoxazole. Resistance to cotrimoxazole was high. Ibadin et al. in Nigeria found 100% sensitivity of *E. coli* to ofloxacin and ciprofloxacin and uniform resistance to cotrimoxazole, amoxicillin and clavulinic acid potentiated amoxicillin in HIV infected adolescents and young adults [16], similar to resistance pattern reported by Asharam et al. in children 0–12 years old both HIV-infected and uninfected [14].

Sterile pyuria was found in 12 children (5.5%), similar to findings by Ibadin et al. (4.1%) in HIV-infected adolescents and young adults [16] but lower than the findings by Shah et al. in India [7]. The children were mainly in WHO clinical stages 3 or 4 (10/12). White cell casts were seen in 9 (4%) and epithelial casts in 3 (1.4%) patients, suggesting pyelonephritis, similar to findings in Uganda [39]. This may suggest widespread renal involvement including tubules in the acute inflammatory process. Ibadin et al. found a higher percentage of white cell casts (14.6%) yet the prevalence of UTI in that cohort was 6.3% suggesting that white cell casts may occur even in the absence of UTI [16]. This may be due to glomerular disease if there is no pyelonephritis or dehydration.

## Conclusions

There is significant burden of urinary tract infections and renal impairment among HIV-infected, ART naive children aged 2–12 years attending the outpatient paediatric HIV clinic at Harare Central Hospital. The renal impairment is more likely to occur in children with advanced WHO clinical stages (3 and 4) and estimation of GFR should be indicated as part of overall assessment in this group. Routine urine dipstick urinalysis should be offered to all HIV-infected children since it is cheap, readily available and allows earlier detection of renal impairment. HIV-infected children with advanced clinical stages 3 and 4 should be monitored for their renal function annually if renal impairment is present at baseline.

### Study limitations

Assessment of orthostatic proteinuria, which was not done, may have affected the prevalence of proteinuria. Microalbuminuria was not assessed and it has been shown to be the first marker of renal disease. Other urinary markers for tubular dysfunction such as phosphorus were beyond the limit of this study. Viral load was not done due to financial constraints but it could have helped to relate the renal abnormalities to viral load. Further investigations such as ultrasound scan for kidney size and echotexture, serum albumin, serum cholesterol levels and triglycerides and renal biopsy were not done though these may not always be available in resource limited settings.

## Abbreviations

ART: Antiretroviral therapy; CI: Confidence interval; CLED: Cystine lactose electrolyte deficient; eGFR: Estimated glomerular filtration rate; HAART: Highly active antiretroviral therapy; HAZ: Height for age z-score; HIV: Human immunodeficiency virus; HIVAN: HIV-associated nephropathy; SD: Standard deviation; UTI: Urinary tract infection; WAZ: Weight for age z-score; WHZ: Weight for height z-score.

## Competing interests

The authors declare that they have no competing interests.

## Authors’ contributions

VD conceived the study, participated in the design, collected data and drafted manuscript. HAM participated in the study design, drafted the manuscript and critically revised it. KJN participated in the study design, critically revised the manuscript. MC participated in the design of the study and performed statistical analysis. ZM carried out the laboratory work, urine microscopy, culture and sensitivity. All authors read and approved the final manuscript.

## Authors’ information

VD Paediatrician MBChB, MMed Paeds, FC Paed (SA).

HAM Consultant Paediatrician, Senior Lecturer, College of Health Sciences, Department of Paediatrics, MBChB, MMed Paeds, MSc Clinical Epidemiology.

KJN Professor of Paediatrics, College of Health Sciences, Department of Paediatrics MBChB, DCH, MRCP, MSc Clinical Epidemiology.

MC Masters Degree in Biostatistics.

ZM Bachelor of Medical Laboratory Sciences Honours Degree (BLMS).

## Pre-publication history

The pre-publication history for this paper can be accessed here:

http://www.biomedcentral.com/1471-2431/13/75/prepub

## Supplementary Material

Additional file 1 **WHO classification of HIV-associated immunodeficiency **[27]. Click here for file
