# Supplementary material for: Renal abnormalities among HIV-infected, antiretroviral naive children, Harare, Zimbabwe: a cross-sectional study
Source: BMC Pediatr. 2013 May 11;13:75. doi: 10.1186/1471-2431-13-75 (PMC3654941; doi:10.1186/1471-2431-13-75)
Supplement: Additional file 1 — WHO classification of HIV-associated immunodeficiency [27]. [file 1471-2431-13-75-S1.doc]

Appendix 1

**WHO classification of HIV-associated immunodeficiency** [27]

| **Classification of HIV-associated immunodeficiency** | **Age related CD4 values** | | | |
| --- | --- | --- | --- | --- |
|  | **≤ 11 months (%)** | **12-35 months (%)** | **36-59 months (%)** | **≥5years (cells/mm^3^)** |
| Not significant | >35 | >30 | >25 | >500 |
| Mild | 30-35 | 25-30 | 20-25 | 350-499 |
| Advanced | 25-29 | 20-24 | 15-19 | 200-349 |
| Severe | <25 | <20 | <15 | <200 or <15% |
